# TRAF3IP3 Is Cleaved by EV71 3C Protease and Exhibits Antiviral Activity

**DOI:** 10.3389/fmicb.2022.914971

**Published:** 2022-06-23

**Authors:** Hui Li, Yunfang Yao, Yu Chen, Shuangling Zhang, Zhi Deng, Wentao Qiao, Juan Tan

**Affiliations:** Key Laboratory of Molecular Microbiology and Technology, Ministry of Education, College of Life Sciences, Nankai University, Tianjin, China

**Keywords:** enterovirus 71, 3C protease, TRAF3IP3, cleavage, virus-host interactions

## Abstract

Enterovirus 71 (EV71) is one of the major pathogens of hand, foot, and mouth disease, which poses a major risk to public health and infant safety. 3C protease (3C^pro^), a non-structural protein of EV71, promotes viral protein maturation by cleaving polyprotein precursors and facilitates viral immune escape by cleaving host proteins. In this study, we screened for human proteins that could interact with EV71 3C^pro^ using a yeast two-hybrid assay. Immune-associated protein TRAF3 Interacting Protein 3 (TRAF3IP3) was selected for further study. The results of co-immunoprecipitation and immunofluorescence demonstrated the interaction between TRAF3IP3 and EV71 3C^pro^. A cleavage band was detected, indicating that both transfected 3C^pro^ and EV71 infection could cleave TRAF3IP3. 87Q-88G was identified as the only 3C^pro^ cleavage site in TRAF3IP3. In Jurkat and rhabdomyosarcoma (RD) cells, TRAF3IP3 inhibited EV71 replication, and 3C^pro^ cleavage partially resisted TRAF3IP3-induced inhibition. Additionally, the nuclear localization signal (NLS) and nuclear export signal (NES) of TRAF3IP3 were identified. The NES contributed to TRAF3IP3 alteration of 3C^pro^ localization and inhibition of EV71 replication. Together, these results indicate that TRAF3IP3 inhibits EV71 replication and 3C^pro^ resists such inhibition *via* proteolytic cleavage, providing a new example of virus-host interaction.

## Introduction

Enterovirus 71 (EV71) is a member of the family *Picornaviridae* and is a genus of *Enteroviruses*. EV71 is the major causative agent of hand, foot, and mouth disease (HFMD) in young children. In 1971, EV71 was first isolated from stool samples of children with neurological diseases in California (Schmidt et al., 1974). Over the last 50 years, there have been several large-scale outbreaks of EV71, including epidemics, worldwide, particularly in the Asia-Pacific region, which seriously threatened public health, especially child health (Ahmad, 2000; Qiu, 2008; Xing et al., 2014). Despite the prevalence of infection, our understanding of the pathogenesis and immune mechanisms of EV71 remains limited, and we lack effective treatments (Ye et al., 2020).

Enterovirus 71 is an RNA virus with a positive single-stranded genome that encodes a polyprotein precursor. Under autocatalytic cleavage and cleavage by two proteases, namely, 2A^pro^ and 3C^pro^, mature viral proteins are produced, including seven nonstructural proteins (2A−3D) and four structural proteins (VP1–VP4) (Shang et al., 2013). EV71 3C^pro^ is a cysteine protease essential for viral replication, where His40, Glu71, and Cys147 are its catalytically active protease sites, and it preferentially cleaves glutamine-glycine (Q-G) and glutamine-serine (Q-S) sites of substrate proteins (Cui et al., 2011; Lei et al., 2011) 3C^pro^ enters the nucleus in its precursor form (3CD' or 3CD), which contains a nuclear localization sequence (NLS) (Sharma et al., 2004). 3C^pro^ mediates immune evasion and disrupts host gene expression by cleaving multiple host proteins to promote virus survival. Innate immune-related proteins, including Toll-like receptor adaptor molecule 1 (TICAM1 or TRIF), interferon regulatory factor (IRF) 7, IRF9, and retinoic acid-inducible gene 1 protein (RIG-I)/IFN-B promoter stimulator 1 (IPS-1) complex, are inhibited or cleaved by 3C^pro^ (Lei et al., 2010, 2011, 2013; Sun et al., 2016). To disrupt host gene transcription, 3C^pro^ cleaves numerous important cellular factors such as the TATA-box-binding protein (TBP), transcription activator p53, cyclic AMP-responsive element-binding protein (CREB), histone H3, and DNA polymerase III (Falk et al., 1990; Clark et al., 1991, 1993; Yalamanchili et al., 1997; Weidman et al., 2001). Screening and identifying host proteins that interact with 3C^pro^, and revealing their cleavage sites and specific mechanisms, will contribute to understanding virus-host interactions during virus infection.

In 2003, tumor necrosis factor receptor-associated factor 3 (TRAF3) interacting protein 3 (TRAF3IP3), also known as TRAF3 interacting Jun N-terminal kinase (JNK)-activating modulator (T3JAM), was first reported to interact with TRAF3, which promotes the specific activation of JNK signaling (Dadgostar et al., 2003). TRAF3IP3 is an immune-associated protein that shows organ and tissue-specific expressions, such as in bone marrow, spleen, and thymus (Dadgostar et al., 2003). TRAF3IP3 is associated with cell maturation and tissue development, is required for the development of T and B cells, and maintains the functional stability of regulatory T cells (Peng et al., 2015; Zou et al., 2015; Yu et al., 2018). TRAF3IP3's subcellular localization is related to its function, appearing mainly in membranous parts, and can be distributed in the endoplasmic reticulum, Golgi apparatus, and mitochondria (Zhu et al., 2019). TRAF3IP3 recruits mitogen/extracellular signal-regulated kinase (MEK) to the Golgi to activate the mitogen-activated protein kinase (MAPK)-extracellular signal-regulated kinase (ERK) pathway (Zou et al., 2015). In the trans-Golgi network, TRAF3IP3 recruits MEK1 and promotes the phosphorylation of ERK and its nuclear translocation (Zhang et al., 2020). Moreover, TRAF3IP3 promotes inflammation by stimulating the translocation of Toll-like receptor 4 (TLR4) into lipid rafts (Li Y. et al., 2019). It has also been reported that TRAF3IP3 induces autophagy and binds to the autophagy regulatory protein autophagy-related 16 like 1 (ATG16L1) (Boada-Romero et al., 2013; Peng et al., 2015). Regarding antiviral innate immune responses, recent studies revealed that TRAF3IP3 exhibits a complex regulatory mechanism. TRAF3IP3 is an important regulator for RIG-I-mitochondrial antiviral signaling (MAVS) protein, in which TRAF3 is recruited to MAVS to promote antiviral innate immunity (Zhu et al., 2019). However, it has also been reported that TRAF3IP3 interacts with TRAF3 and TANK-binding kinase 1 (TBK1) and promotes degradation of ubiquitinated TBK1, thereby negatively regulating cytosolic RNA-induced antiviral signaling (Deng et al., 2020). Currently, the functions of TRAF3IP3 are still underreported, especially its contribution to antiviral events.

In this study, we used EV71 3C^pro^ as the bait in a yeast two-hybrid experiment, which captured TRAF3IP3 from a human cDNA library. The interaction between TRAF3IP3 and 3C^pro^ was confirmed, and TRAF3IP3 was then identified as a substrate of EV71 3C^pro^. TRAF3IP3 87Q-88G was the only site that was cleaved by 3C^pro^. Furthermore, TRAF3IP3 showed antiviral activity in Jurkat and rhabdomyosarcoma (RD) cells, making this the first report of TRAF3IP3 inhibition of an enterovirus. EV71 3C^pro^ resisted these antiviral effects by cleaving TRAF3IP3. Subsequently, the nuclear localization signal and nuclear export signal (NES) of TRAF3IP3 were identified. The NES of TRAF3IP3 maintained its localization in the cytoplasm, hindered 3C^pro^ entry into the nucleus, and contributed to the inhibition of EV71 replication. In this study, we revealed a novel relationship between EV71 and host protein TRAF3IP3 and expanded the study of host antiviral mechanisms.

## Materials and Methods

### Plasmids and Transfection

Professor Zhiyong Lou (Tsinghua University) kindly donated the EV71 infectious clone pSVA-EV71. The *TRAF3IP3* cDNA was synthesized by Genewiz Biotechnology Co., Ltd. (Suzhou, China) and then ligated into the eukaryotic expression vectors pCMV-3×HA and pQCXIP with an N-terminal Flag tag. The 3C protease cDNA was PCR amplified from pSVA-EV71 and ligated into pEGFP-C3 (EGFP, enhanced green fluorescent protein) and pQCXIP together with an N-terminal Myc tag. 3C-C147S (3C with Cys at position 147 mutated to Ser) was constructed by PCR site-directed mutagenesis from 3C^pro^ using the following primers: forward, 5′-GTACTGCGTAGTCATACGTACGATACGTG-3′ and reverse, 5′-CACGTATCGTACGTATGACTACGCAGTAC-3′. Cells were transfected using Lipofectamine 3000 (Thermo Fisher Scientific, Waltham, MA, USA) or polyethyleneimine (PEI; Polysciences, Warrington, PA, USA) according to the manufacturer's protocol.

### Cell Culture and Virus Infection

The HeLa, HEK293T, and human muscle RD cells were maintained in Dulbecco's modified Eagle's medium (DMEM; Gibco, Grand Island, NY, USS) supplemented with 10% fetal bovine serum (FBS; Gibco) and penicillin-streptomycin. Jurkat cells were maintained in Roswell Park Memorial Institute (RPMI) 1640 medium (Gibco) supplemented with 10% FBS, penicillin-streptomycin, and 2 mM L-glutamine. Cells were cultured in a humidified atmosphere containing 5% CO_2_ at 37°C. The SK-EV006/Malaysia/97 strain of EV71 (GenBank number AB469182.1) was produced in RD cells. The infectious clone pSVA-EV71 was linearized using *Sal* I, and transcribed *in vitro* into RNA, followed by Lipofectamine 3000-mediated transfection into RD cells for EV71 packaging. The MEGAscript T7 High Yield Transcription Kit (Thermo Fisher Scientific) was used to perform *in vitro* transcription. Virus preparation, titration, and infection methods were as previously described (Li et al., 2022).

### Western Blotting Analysis

Cells were collected and rinsed once using ice-cold phosphate-buffered saline (PBS), before being lysed in the buffer comprising 50 mM Tris (pH 7.4), 150 mM NaCl, 2 mM EDTA, 0.5% sodium deoxycholate, 1% NP-40, and protease inhibitor cocktail (Roche, Basel, Switzerland). The lysate was centrifuged, and the supernatant was retained and added with loading buffer before being boiled for 10 min at 100°C. SDS-PAGE was used to resolve the proteins in the samples, which were transferred electrophoretically onto polyvinylidene fluoride membranes (GE Healthcare, Chicago, IL, USA). Phosphate-buffered saline–Tween 20 (PBST) with 5% non-fat milk was used to block the membranes for 45 min at room temperature. The indicated primary antibodies were then used to probe the blot for 90 min at room temperature or overnight at 4°C. The primary antibodies recognizing HA and Flag were purchased from Sigma (St. Louis, MO, USA); the anti-Myc, anti-GFP, and anti-Tubulin antibodies were from Santa Cruz Biotechnology (Santa Cruz, CA, USA). The anti-TRAF3IP3 (immunogen 300–551 aa), anti-3C^pro^, and anti-VP1 antisera were obtained in our laboratory by immunizing mice with purified recombinant proteins. After incubation with goat anti-rabbit or mouse horseradish peroxidase (HRP)-conjugated secondary antibodies (Santa Cruz Biotechnology), the Immobilon Western Chemiluminescent HRP Substrate (Millipore, Billerica, MA, USA) was used to treat the membranes for chemiluminescent detection, which was visualized using the Junyi Capture e610 System (JUNYI, Beijing, China).

### Yeast Two-Hybrid Assay

The 3C^pro^ cDNA was cloned into pGBKT7 as the bait. The construct was transformed into *Saccharomyces cerevisiae* AH109. The transformed yeast cells were lysed to detect bait expression and then spread onto SD/-Trp and SD/-His plates to test auto-activation. After that, in a sterile flask, 1 ml of Mate & Plate Library™ (Universal Human Normalized, cat. no. 630480, Takara, Dalian, China) was mixed with 4–5 ml of the bait strain and then added with 45 ml of 2× yeast potato dextrose (YPD) liquid medium containing 50 μg/ml kanamycin. The mixture was incubated for 20–30 h at 30°C with slow shaking. Cell pellets were collected by centrifugation, and the pelleted cells were resuspended in 10 ml 0.5× YPD/kanamycin liquid medium. Cultures were spread on SD/-Leu/-Trp/-His/-Ade agar plates at 200 μl per 150 mm and incubated at 30°C for 3–5 days. Single colonies were picked and plated on SD/-Leu/-Trp/-His/-Ade/X-α-Gal agar plates. Plasmids from blue colonies were extracted and transformed into *E. coli* DH5α. The extracted plasmids were identified by *Xho* I and *Nde* I digestion and sequencing. The sequencing results were BLAST searched at GenBank in the National Center for Biotechnology Information (NCBI).

### Co-immunoprecipitation

The indicated plasmids were transfected into HEK293T cells for 48 h and then lysed using ultrasound in Western blotting lysis buffer. Cell lysates were then incubated with corresponding antibodies for 2 h at 4°C on a rotator, followed by incubation with protein A agarose beads (Santa Cruz) at 4°C for 3 h on a rotator. The mixture was rinsed six times using a buffer containing 400 mM NaCl, 1% Triton X-100, and 1% sodium dodecyl sulfonate (SDS). The final pellets were separated using SDS-PAGE followed by Western blotting analysis.

### Immunofluorescence Assay

The HeLa cells that proliferated on coverslips in a 12-well plate to 80% confluence were transfected with the indicated plasmids. After washing using ice-cold PBS, the cells were fixed using 4% paraformaldehyde in PBS at room temperature for 10 min and permeabilized using 0.1% Triton X-100 in PBS for 10 min. Then, blocking buffer [containing 50% FBS, 5% non-fat milk, 3% bovine serum albumin (BSA), 0.5% NaN_3_, 0.1% Triton X-100 in PBS] was incubated with the fixed cells for 2 h. The cells were then incubated with anti-HA or anti-Myc antibodies for 2 h. After washing with 0.1% Triton X-100 in PBS four times, the cells were incubated with fluorescein isothiocyanate (FITC) or tetramethylrhodamine isothiocyanate (TRITC)-conjugated goat secondary antibodies for 40 min. Cellular nuclei were stained with 0.2 μg/ml 4',6-diamidino-2-phenylindole (DAPI) for 10 min. The samples were imaged under an Olympus IX71 fluorescence microscope (Olympus, Tokyo, Japan).

### *In vitro* Cleavage Assay

The cDNA of EV71 3C protease or mutant 3C-C147S was cloned into vector pET-28a. The plasmids were transformed into *Escherichia coli* BL21 (DE3) and induced with 1 mM isopropyl β-d-1-thiogalactopyranoside for expression. After the cells were harvested and lysed, the lysate was loaded onto a 15 ml Ni-nitrilotriacetic acid (NTA) column equilibrated in lysis buffer. The column was eluted using 200 mM imidazole in lysis buffer to obtain purified protein 3C^pro^ or the C147S mutant. HEK293T cells were transfected with the *TRAF3IP3* expression plasmid. At 48 h after transfection, the cells were harvested. The lysate was centrifuged at 12,000 rpm for 10 min at 4°C. The supernatant was mixed with purified 3C^pro^ in cleavage buffer (200 mM NaCl and 50 mM pH 7.0 Tris-HCl) and incubated for 90 min at 30°C. The reaction was stopped by adding loading buffer and boiling for 15 min at 100°C. The cleavage effect was assessed using Western blotting.

### Lentivirus-Based Short Hairpin RNA (shRNA) Knockdown and Stable Expression

Double-stranded oligonucleotides corresponding to the shRNA target sequences were inserted into vector pSIREN-RetroQ. The target sequences comprised: shNC (negative control lacking a specific target in the cells) 5′-GACAGAACCAGAGGATAGA-3′, and shTRAF3IP3 5′-GATCCGGGACAGCTTAATGAAGACTTCAAGAGAGTCTTCATTAAGCTGTCCCTTTTTTACGCGTG-3′. Then, 1 μg of pMLV-Gag-Pol, 0.5 μg of pVSV-G, and 1 μg of pSIREN-RetroQ-shNC or shTRAF3IP3 were transfected into HEK293T cells. At 48 h after transfection, we harvested the viral supernatants and used them to infect Jurkat cells. At 48 h after infection, the Jurkat cells were subcultured in a selective medium with 2 μg/ml puromycin (Sigma). The efficiency of knockdown was determined using Western blotting.

The *TRAF3IP3* cDNA was cloned into vector pQCXIP (introducing a Flag tag in the upstream primer). Then, 1 μg of pMLV-Gag-Pol, 0.5 μg of pVSV-G, and 1 μg pQCXIP vector or pQCXIP-TRAF3IP3 (N-Flag) were transfected into HEK293T cells. The procedure for infection and selection of Jurkat cells was the same as for the knockdown described above. The effect of stable expression was detected by Western blotting.

## Results

### TRAF3IP3 Interacts With EV71 3C^pro^

**Figure 1:**
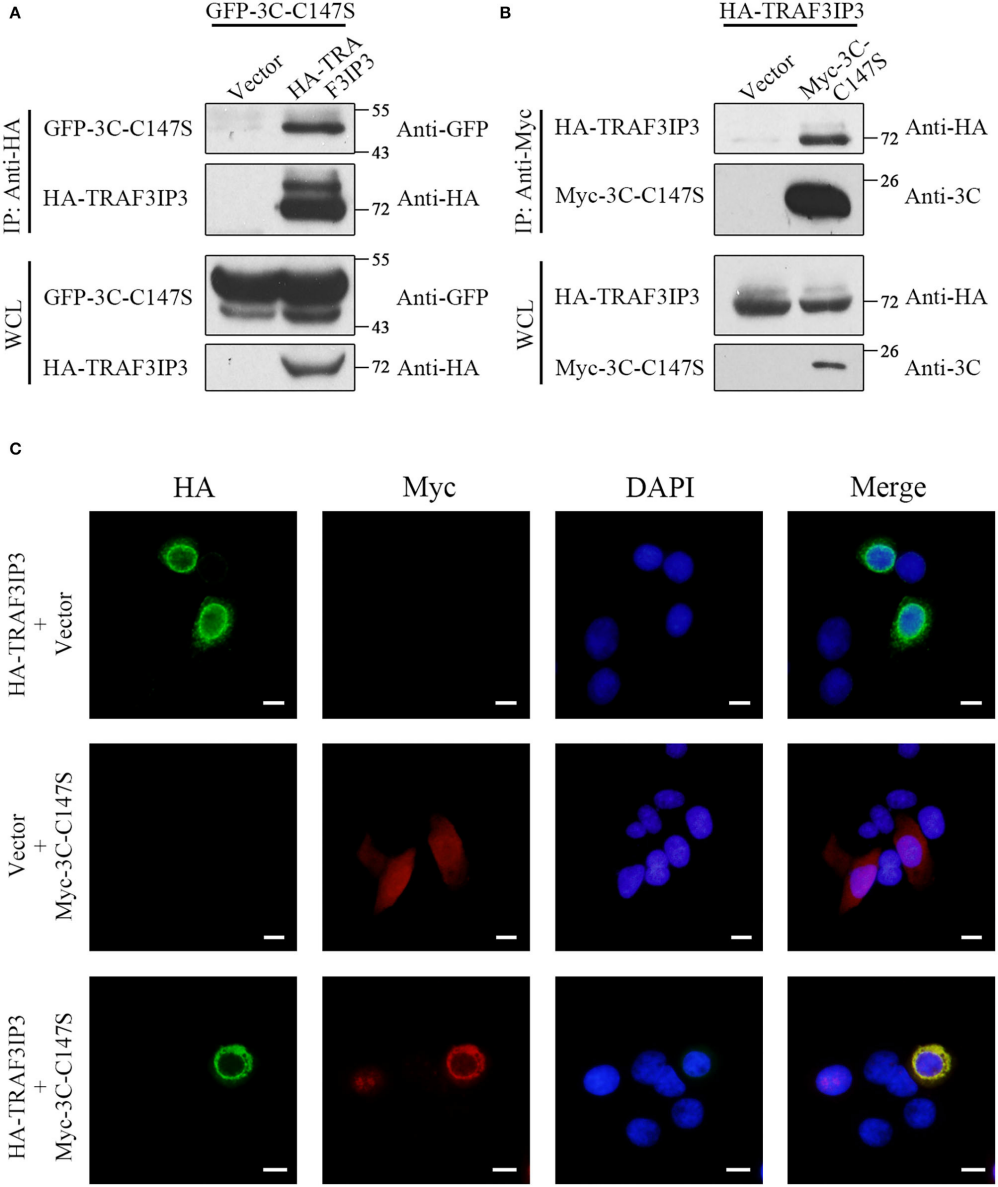
TRAF3IP3 interacts with EV71 3C^pro^. **(A)** HEK293T cells were co-transfected with GFP-3C-C147S and HA-TRAF3IP3. Cells were harvested 48 h after transfection and co-immunoprecipitated using anti-HA antibodies, and protein interactions were detected using Western blotting. WCL, whole-cell lysate. **(B)** HEK293T cells were co-transfected with HA-TRAF3IP3 and Myc-3C-C147S. Anti-Myc antibodies were used for co-immunoprecipitation, and other operations were the same as in **(A)**. **(C)** HA-TRAF3IP3 or Myc-3C-C147S were transfected into HeLa cells. 48 h later, the cells were fixed and protein subcellular localizations were observed using immunofluorescence. Scale bars = 10 μm.

Proteins that might interact with EV71 3C^pro^ were screened initially using a universal human library and the yeast two-hybrid (Y2H) system, with 3C^pro^ as the bait. We obtained 205 positive clones by nutritional and colorimetric selection. We identified nearly 60 proteins in the Y2H results (Supplementary Table 1), and 5 positive clones contained *TRAF3IP3* cDNA fragments. Considering the high false-positive rate in Y2H screening, co-immunoprecipitation (co-IP) was performed to confirm the interaction between 3C^pro^ and TRAF3IP3. We used C147S, a 3C mutant lacking protease activity, as an alternative of 3C^pro^ to exclude the influence of cleavage. HEK293T cells were co-transfected with TRAF3IP3 and C147S expression plasmids, cultured, and lysed, followed by co-IP using anti-Myc or anti-HA antibodies from the lysate. The interaction between C147S and TRAF3IP3 was observed (Figures 1A,B). In addition, immunofluorescence analysis revealed co-localization of C147S and TRAF3IP3 (Figure 1C). These results suggested that EV71 3C^pro^ interacts with TRAF3IP3.

### TRAF3IP3 Is a Substrate of EV71 3C^pro^ Protease

**Figure 2:**
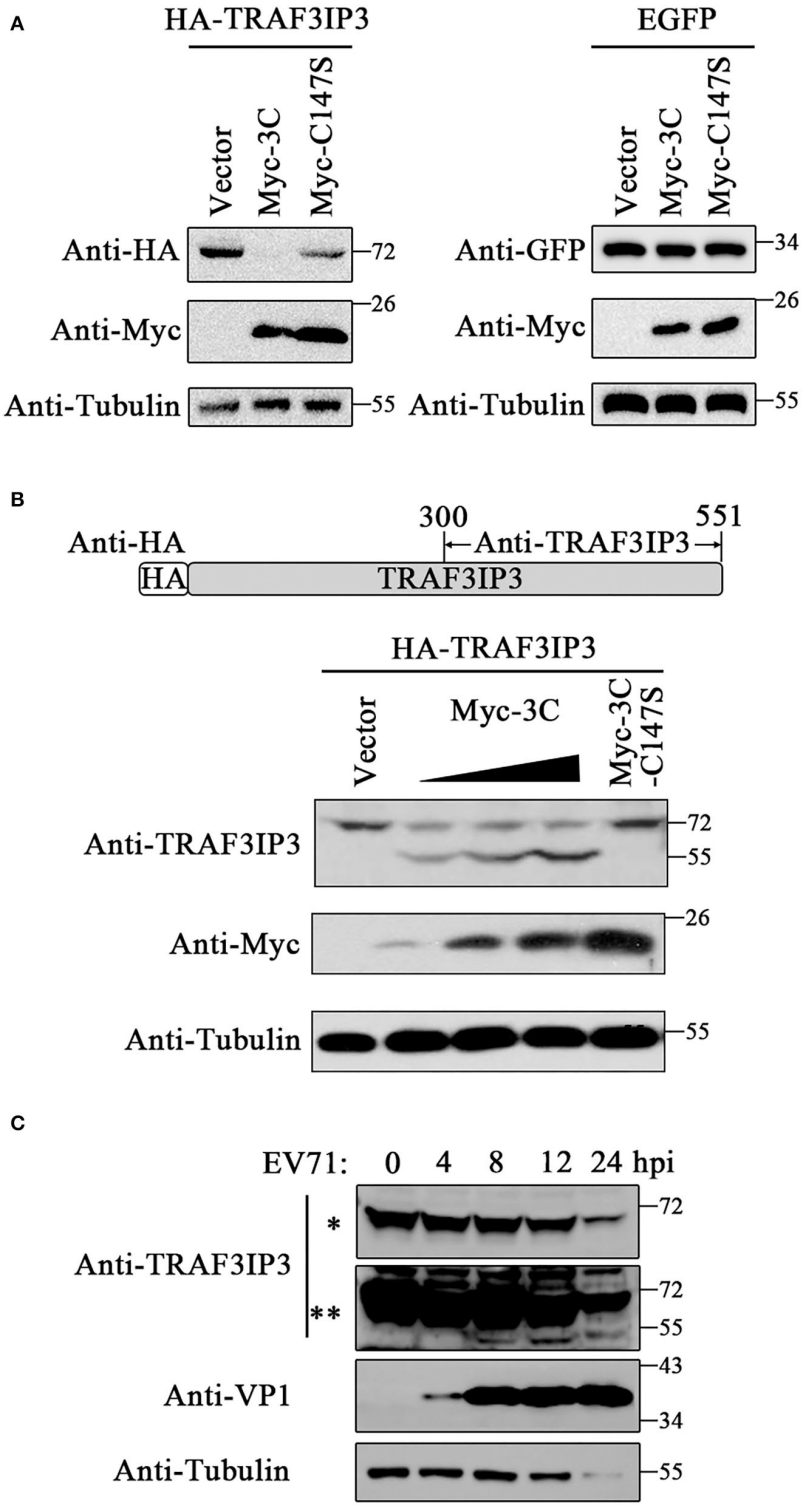
TRAF3IP3 is the cleavage substrate for 3C^pro^. **(A)** HA-TRAF3IP3 or GFP was co-transfected with 3C^pro^ or C147S into HEK293T cells. Cells were harvested 48 h later for Western blotting. **(B)** HEK293T was co-transfected with HA-TRAF3IP3 and a gradient of Myc-3C, with C147S as a non-cleavable control. The cleavage bands were detected using the antibody recognizing the C-terminus of TRAF3IP3. **(C)** EV71 at an MOI of 10 was used to infect into Jurkat cells for 0, 4, 8, 12, and 24 h, and the cleavage of endogenous TRAF3IP3 was detected using Western blotting. * and ** represent short and long exposures, respectively.

Previous studies have determined that 3C^pro^ could cleave various host proteins during the EV71 life cycle depending on its protease activity. To investigate whether EV71 3C^pro^ cleaves TRAF3IP3 during their interaction, TRAF3IP3 was co-expressed with 3C^pro^ or C147S in HEK293T cells, respectively. The amount of the full-length TRAF3IP3 decreased in the presence of 3C^pro^, but not with C147S (Figure 2A left). 3C^pro^ could not decrease the level of EGFP (Figure 2A right), indicating that the 3C^pro^-induced reduction in TRAF3IP3 was specific. Anti-TRAF3IP3 antibodies were prepared by immunizing mice with a purified fragment of TRAF3IP3 (300–551 amino acids) (Figure 2B). 3C^pro^ cleaved TRAF3IP3 in a concentration-dependent manner, and a cleavage band of approximately 55 kDa was detected using the C-terminal anti-TRAF3IP3 polyclonal antibodies (Figure 2B). To observe the effect of 3C^pro^ during EV71 infection, EV71 at a multiplicity of infection (MOI) of 10 was used to infect Jurkat cells, which naturally express high levels of TRAF3IP3, and the cells were collected at the indicated time points. The amount of the full-length TRAF3IP3 decreased, while the level of the cleavage band increased, time-dependently (Figure 2C). Thus, TRAF3IP3 is a substrate of EV71 3C^pro^ protease during viral infection.

### EV71 3C^pro^ Protease Cleaves TRAF3IP3 at 87Q-88G

**Figure 3:**
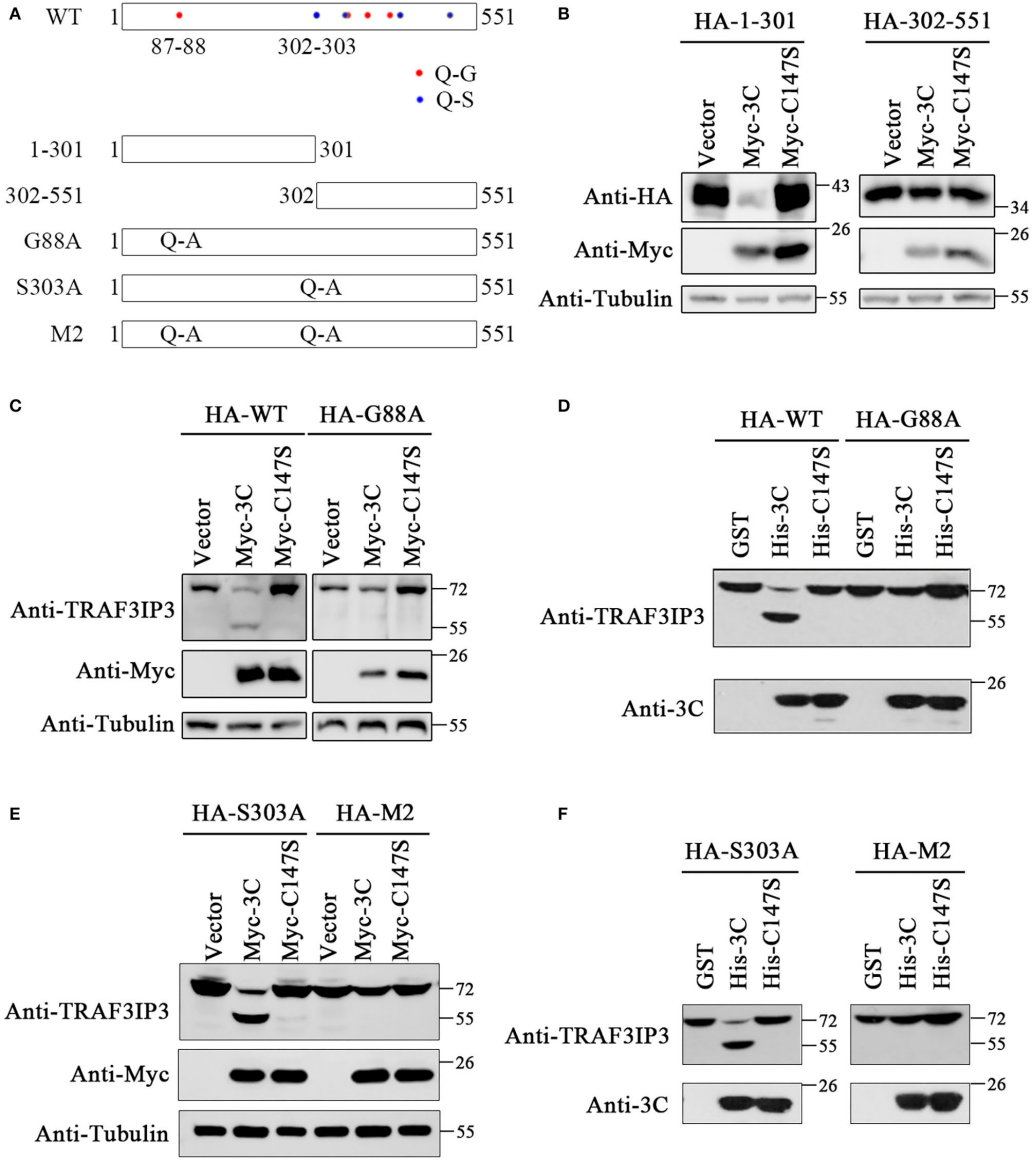
The 87Q-88G is the only site in TRAF3IP3 that is cleaved by 3C^pro^. **(A)** Schematic representation of the potential 3C^pro^ cleavage sites in TRAF3IP3. **(B)** HEK293T cells were transfected with HA-TRAF3IP3 1–301 or 302–551 and Myc-3C. Forty-eight hours later, Western blotting was used to detect the proteins. **(C)** HA-TRAF3IP3 wild-type or G88A mutant was co-transfected with Myc-3C or mutant C147S into HEK293T cells. Cells were harvested 48 h after transfection for Western blotting analysis. **(D)** HEK293T cells were transfected with HA-TRAF3IP3 wild-type or G88A mutant. Cells were harvested 48 h after transfection and lysates were incubated with purified 3C^pro^ or mutant 3C-C147S for 90 min at 30°C. Loading buffer was added to stop the reaction, followed by Western blotting detection of proteins. **(E)** HA-TRAF3IP3 single-mutant S303A or double-mutant G88A S303A (M2) were co-transfected with Myc-3C or mutant C147S into HEK293T cells. Cells were harvested 48 h after transfection for Western blotting. **(F)** HA-TRAF3IP3-S303A or M2 was transfected into HEK293T cells. The rest of the experiment was the same as the *in vitro* cleavage in **(D)**.

To identify the EV71 3C^pro^ cleavage sites, we analyzed the TRAF3IP3 amino acid sequence. According to previous research, EV71 3C^pro^ preferentially cleaves target proteins at glutamine-glycine (Q-G) and glutamine-serine (Q-S) sites (Sun et al., 2016). There are eight potential cleavage sites in TRAF3IP3: 87Q-88G, 302Q-303S, 347Q-348S, 351Q-352G, 382Q-383G, 417Q-418G, 423Q-424S, and 514Q-515S (Figure 3A). To be more efficient, two truncated proteins of TRAF3IP3 1–301 aa and 302–551 aa were constructed. They were then co-transfected with 3C^pro^ or C147S into HEK293T cells, respectively. The level of TRAF3IP3 1–301 was reduced by 3C^pro^, but 302–551 was not (Figure 3B), indicating that the cleavage mainly occurred in the N-terminal half of the protein. In fragment 1–301, there was only one potential site, 87Q-88G. To verify whether 87Q-88G is the cleavage site recognized by 3C^pro^, we constructed a single mutation G88A. Vectors expressing wild-type TRAF3IP3 and the G88A mutant were co-transfected with 3C^pro^, and the result of Western blotting showed that the G88A mutation protected full-length TRAF3IP3 from cleavage (Figure 3C). *In vitro*, consistent results were obtained using purified 3C^pro^ or C147S (Figure 3D). Thus, we identified 87Q-88G as a 3C^pro^ cleavage site in TRAF3IP3. We considered the possibility of other sites. The site 302Q-303S was the first amino acid of 302–551. Single-mutant S303A and double-mutant G88A S303A (M2) were used to confirm whether 302Q-303S was also a cleavage site. The co-transfection (Figure 3E) and *in vitro* (Figure 3F) analyses showed that mutation M2 did not reduce the production of cleavage band, but the mutation S303A did. In summary, 3C^pro^ cleaves TRAF3IP3 at 87Q-88G, but not at 302Q-303S.

### 3C^pro^ Cleavage of TRAF3IP3 Partially Alleviates Its Inhibition of EV71 Replication

**Figure 4:**
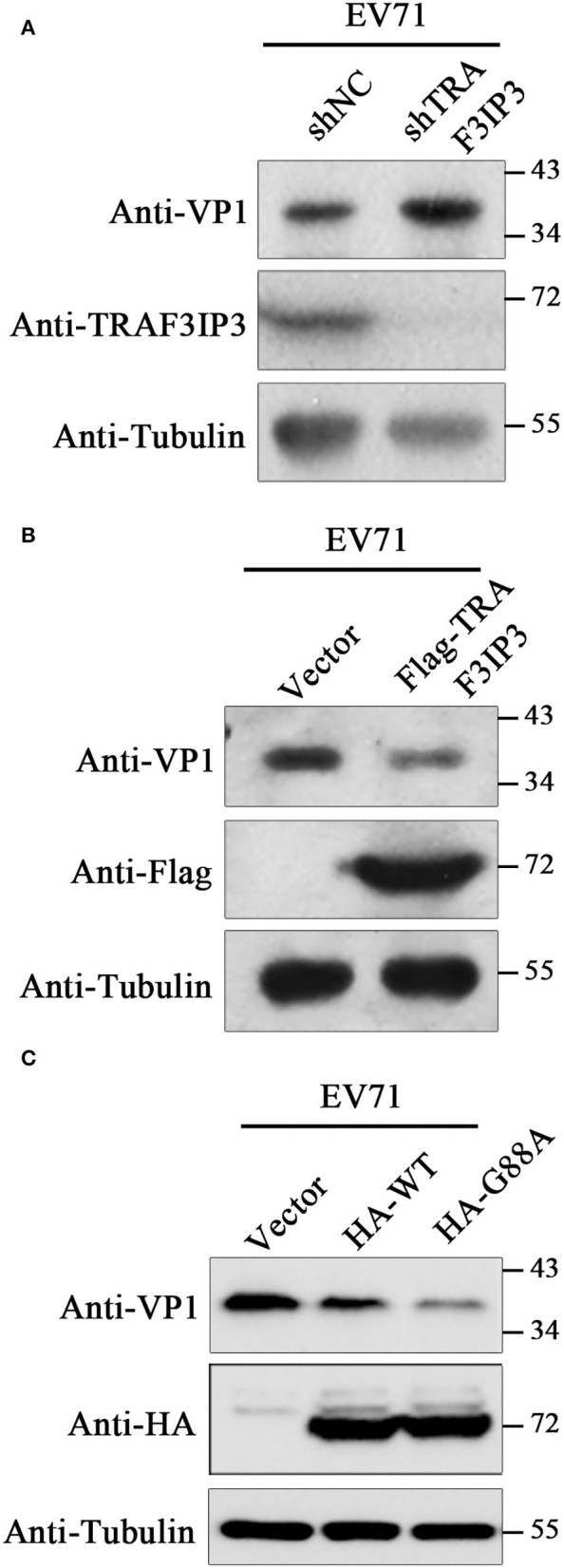
TRAF3IP3 inhibits EV71 replication. **(A)** Jurkat cells were first infected with lentiviruses expressing negative control shRNA (shNC) or an shRNA targeting *TRAF3IP3* (shTRAF3IP3). Cells were selected with puromycin until the knockdown effect stabilized. Jurkat cells were harvested for Western blotting at 12 h after infection with 1 MOI EV71. **(B)** Jurkat cells were first infected with lentiviruses expressing Flag-TRAF3IP3 or vector. The rest of the experiment was the same as **(A)**. **(C)** HA-TRAF3IP3 wild-type or G88A mutant was transfected into RD cells, followed by infection with EV71 at 1 MOI at 36 h after transfection. Twelve hours later, the cells were collected for Western blotting.

To investigate the effects of TRAF3IP3 on EV71 replication, EV71 at an MOI of 1 was used to infect *TRAF3IP3* knockdown Jurkat cells, which were harvested at 12 h after infection for Western blotting analysis. EV71 produced more viral protein in *TRAF3IP3* knockdown Jurkat cells than in wild-type Jurkat cells (Figure 4A). The same experiment was carried out using *TRAF3IP3* overexpressing Jurkat cells. The result showed that overexpression of *TRAF3IP3* suppressed EV71 replication (Figure 4B). To determine how cleavage of TRAF3IP3 affected EV71 replication, vectors expressing wild-type TRAF3IP3 or the cleavage-resistant mutant G88A were transfected into RD cells, followed by infection with EV71 at 1 MOI. At 12 h post-infection, Western blotting of cell lysates showed that wild-type TRAF3IP3 inhibited the replication of EV71, while G88A had a stronger inhibitory effect (Figure 4C). This stronger anti-virus effect of the cleavage-resistant mutant indicated that EV71-mediated cleavage partially alleviates TRAF3IP3-induced viral suppression.

### The NES of TRAF3IP3 Contributes to the Antiviral Effect

**Figure 5:**
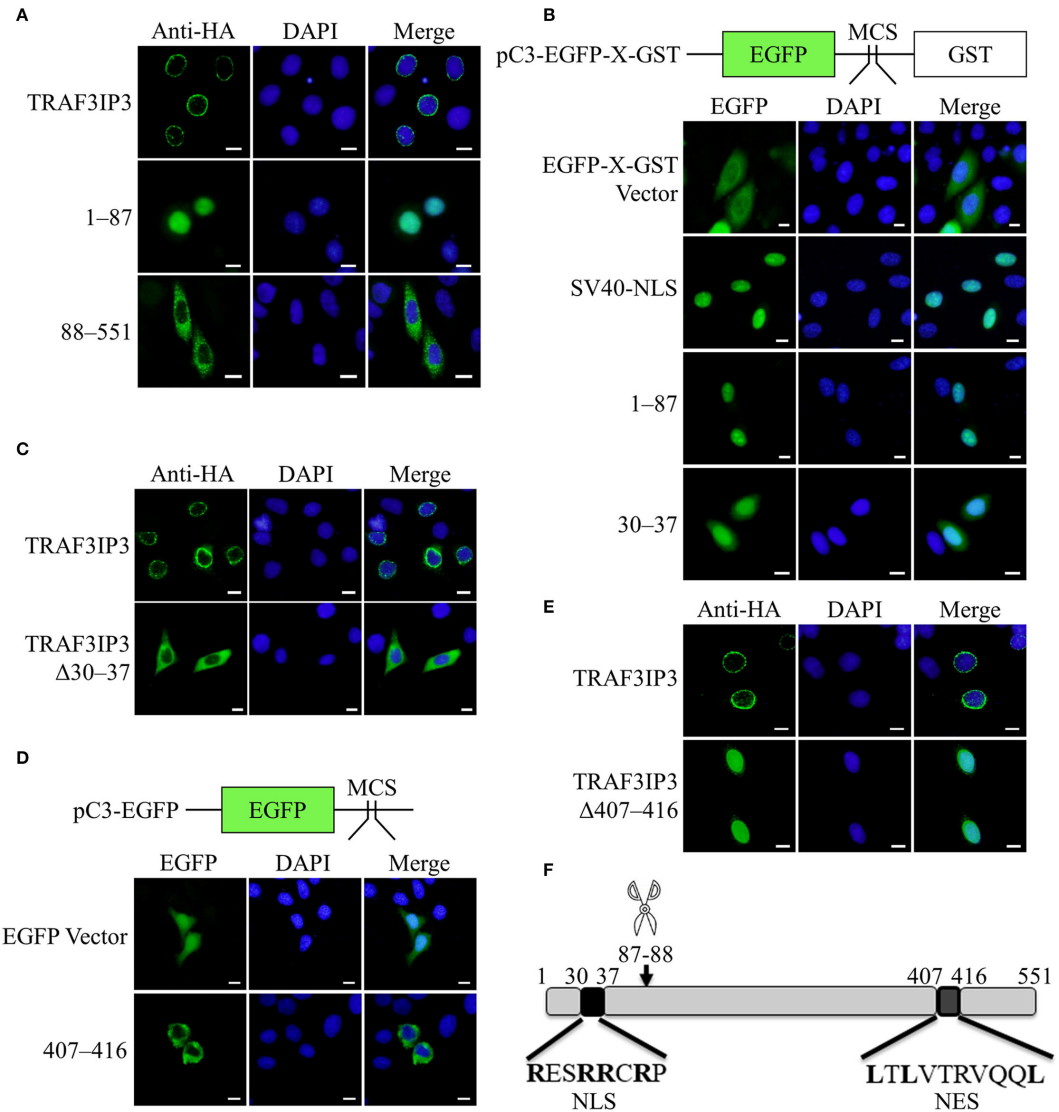
Identification of the NLS and NES of TRAF3IP3. The subcellular localization of the protein was detected using immunofluorescence analysis. Cells were fixed 48 h after transfection. Scale bars represent 10 μm. **(A)** HeLa cells were transfected with HA-TRAF3IP3, HA-TRAF3IP3 1–87, or HA-TRAF3IP3 88–551. **(B)** Schematic representation of the pC3-EGFP-X-GST vector. HeLa cells were transfected with EGFP-X-GST vector, EGFP-SV40 NLS-GST, EGFP-TRAF3IP3 1–87-GST, or EGFP-TRAF3IP3 30–37-GST. **(C)** HeLa cells were transfected with HA-TRAF3IP3 or HA-TRAF3IP3 Δ30–37. **(D)** Schematic representation of the pC3-EGFP vector. HeLa cells were transfected with EGFP vector or EGFP-TRAF3IP3 407–416. **(E)** HeLa cells were transfected with HA-TRAF3IP3 or HA-TRAF3IP3 Δ407–416. **(F)** Schematic representation of the NLS and NES of TRAF3IP3.

The results in Figure 1C showed that TRAF3IP3 brings EV71 3C^pro^ out of the nucleus, prompting us to consider whether the location of the cleavage products affects EV71 replication. HeLa cells were transfected to overexpress TRAF3IP3 or the cleavage products 1–87 and 88–551. Immunofluorescence results showed that the locations of the three proteins were different: TRAF3IP3 was located around the nucleus, fragment 1–87 was located in the nucleus, and fragment 88–551 was located in the cytoplasm (Figure 5A). We assumed that fragment 1–87 contained an NLS and fragment 88–551 contained an NES. To confirm whether fragment 1–87 entered the nucleus through free diffusion due to its low molecular weight, it was inserted into the dual-tag vector pC3-EGFP-X-GST (Ma et al., 2014). The detection of EGFP fluorescence signal indicated that 1–87 allowed EGFP-X-GST to enter into the nucleus similar to the positive control, SV40-NLS (Figure 5B). An NLS generally contains 4–8 amino acids, the core is mostly positively charged R or K, and the two ends are P or G (Kalderon et al., 1984). Using cNLS Mapper online prediction, the NLS sequence was located at ^30^RESRRCRP^37^ of TRAF3IP3. Retaining only residues 30–37 of TRAF3IP3 could also allow EGFP-X-GST to enter into the nucleus (Figure 5B). When residues 30–37 were deleted from TRAF3IP3, the results showed that compared with wild type, Δ30–37 tended to be located in the cytoplasm (Figure 5C). These results support the hypothesis that residues 30–37 are the NLS of TRAF3IP3. NetNES was used to predict that the NES of TRAF3IP3 was ^407^LTLVTRVQQL^416^ (la Cour et al., 2004). Residues 407–416 were inserted into the vector pC3-EGFP, and the detection of the EGFP signal indicated that the presence of residues 407–416 caused EGFP, which originally diffused into the nucleus, to exit from the nucleus (Figure 5D). When residues 407–416 were deleted from the total length of TRAF3IP3, the protein remained in the nucleus rather than being distributed around the nucleus (Figure 5E). These results illustrated that residues 407–416 form the NES of TRAF3IP3.

The summary diagram in Figure 5F shows that 87Q-88G of TRAF3IP3 is the cleavage site of EV71 3C^pro^, the N-terminal sequence ^30^RESRRCRP^37^ is an NLS, and the C-terminal sequence ^407^LTLVTRVQQL^416^ is an NES.

**Figure 6:**
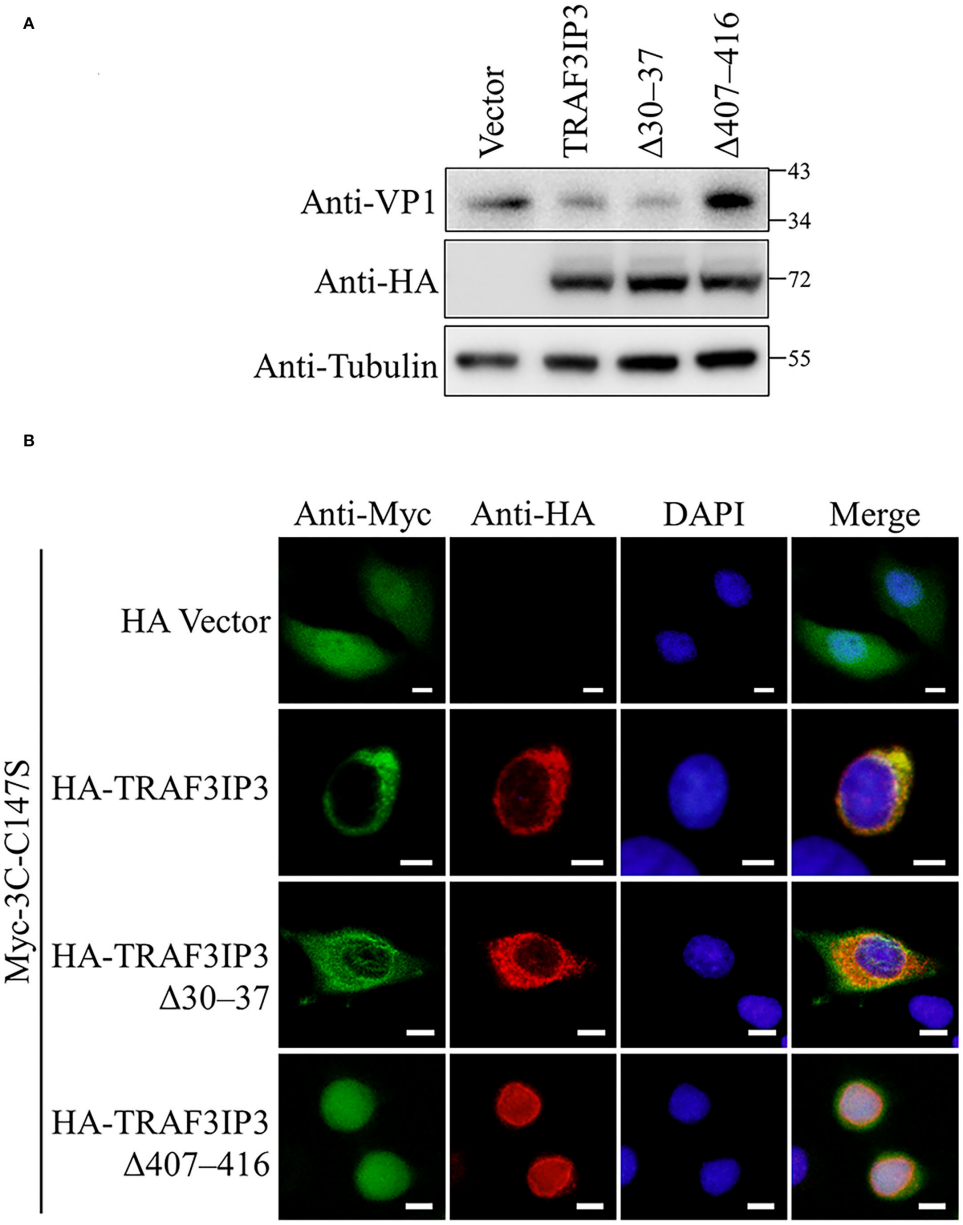
The NES of TRAF3IP3 contributes to the inhibition of EV71. **(A)** HA-TRAF3IP3 wild-type or deletion mutants were transfected into RD cells, followed by infection with EV71 at an MOI of 1 at 36 h post-transfection. Twelve hours later, the cells were collected for Western blotting. **(B)** HA-TRAF3IP3 wild-type or deletion mutants and Myc-3C-C147S were co-transfected into HeLa cells. Cells were fixed for immunofluorescence after 48 h. Scale bars represent 10 μm.

The effects of the NLS and NES on the inhibition of EV71 replication by TRAF3IP3 were tested. RD cells were transfected to overexpress TRAF3IP3, Δ30–37, or Δ407–416, followed by infection with EV71 at 1 MOI for 12 h. Subsequent Western blotting showed that TRAF3IP3 and Δ30–37 inhibited EV71 replication, whereas Δ407–416 did not (Figure 6A), indicating that residues 407–416 are necessary for TRAF3IP3 to inhibit EV71 replication. To explore the impact on the location of 3C^pro^, HeLa cells were co-transfected with TRAF3IP3, Δ30–37, or Δ407–416 and 3C^pro^. Immunofluorescence assays revealed that TRAF3IP3 and Δ30–37 could retain 3C^pro^ in the cytoplasm, but Δ407–416 could not (Figure 6B). These results indicated that the NES of TRAF3IP3 contributes to the inhibition of EV71 replication and changes the localization of 3C^pro^.

## Discussion

As a viral pathogen, EV71 relies on host cells for replication and competes with the host throughout its life cycle. The 3C protease is one of the non-structural proteins that regulates multiple signal pathways of the host *via* its protease activity, to gain an advantage in virus-host competition (Wen et al., 2020). It has been reported that 3C^pro^ cleaves a variety of host proteins (Wang et al., 2015; Lei et al., 2017; Li et al., 2017; Li M. L. et al., 2019), revealing the molecular mechanism of interference with host cells. The results of this study revealed that TRAF3IP3 is a target of EV71 3C^pro^. TRAF3IP3 inhibits EV71 virus replication, and EV71 antagonizes TRAF3IP3 by cleaving it.

The important functions of 3C^pro^ have led to it becoming a significant molecular target for antiviral therapy (Ma et al., 2016). Identifying 3C^pro^ interacting proteins helps to understand the impact of 3C^pro^ on host cells and eliminate harm from the virus. In this report, Y2H technology was used to screen host proteins that interact with EV71 3C^pro^. In our previous work, we found that 3C^pro^ cleaves PinX1, which was also detected by the Y2H assay (Number 54 in Supplementary Table 1), to promote cell apoptosis and EV71 release (Li et al., 2017). In this research, TRAF3IP3, which showed 5 positive clones in the screening, was identified as a cleavage substrate of EV71 3C^pro^ for the first time, and the exact cleavage site was revealed. Co-IP and immunofluorescence experiments confirmed the results of Y2H assay: TRAF3IP3 interacts with EV71 3C^pro^, which satisfies one of the necessary conditions for enzymes and substrates. Through cell transfection, virus infection, and *in vitro* cleavage experiments, we confirmed that TRAF3IP3 is a cleavage substrate of EV71 3C^pro^ and that 87Q-88G is the only cleavage site in TRAF3IP3. According to previous reports, 3C^pro^ identifies and cleaves proteins at Q-G sites, particularly sites with a AxxQ/G sequence, where x is any amino acid and the cleavage site is indicated with a slash (Nicklin et al., 1988; Guo et al., 2014). There are four Q-G sites in TRAF3IP3, but only 87Q-88G complies with the consensus sequence (^84^AREQ/G^88^). Thus, we identified a classic 3C^pro^ cleavage site in TRAF3IP3.

This is the first report that TRAF3IP3 inhibits an enterovirus. Since TRAF3IP3 was discovered in 2003 (Dadgostar et al., 2003), there have been few reports on its function. TRAF3IP3 is related to the cell development of the mouse thymus and spleen and is highly expressed in immune organs and tissues (Dadgostar et al., 2003; Peng et al., 2015; Zou et al., 2015). As for virus-related reports, Zhu et al. reported that the activation of TBK1-interferon regulatory factor 3 (IRF3) and downstream type I interferon production are mediated by TRAF3IP3-induced recruitment of TRAF3 to MAVs (Zhu et al., 2019). However, Deng et al. (2020) proposed that TRAF3IP3 weakens the type I interferon response by promoting the ubiquitination and degradation of TBK1. TRAF3IP3 seems to have a complex positive and negative regulation in the antiviral innate immune response. Our results indicated that TRAF3IP3 inhibits EV71 replication. In fact, EV71 destroys the interferon pathway through a variety of mechanisms, resulting in poor induction of interferon in infected cells, which is considered to be a method to combat the innate immune system (Rasti et al., 2019). We propose a more direct antiviral mechanism: TRAF3IP3's interaction with 3C^pro^ interferes with its localization, thereby inhibiting the normal function of 3C^pro^. Similar to its antagonism toward interferon, EV71 also partially resists inhibition by TRAF3IP3, which was reflected by the fact that the G88A mutant could not be cleaved by 3C^pro^ and thus had a stronger antiviral effect than wild-type TRAF3IP3.

The function of TRAF3IP3 is related to its localization. TRAF3IP3 mediates B-Raf proto-oncogene, serine/threonine kinase (BRAF)-MEK-ERK signaling by recruiting MEK to the Golgi (Zou et al., 2015). During Sendai virus infection, TRAF3IP3 accumulates on mitochondria (Zhu et al., 2019). In T reg cells, the mechanistic target of rapamycin (mTORC1) signaling is regulated by TRAF3IP3-promoted protein phosphatase 2 catalytic subunit alpha (PP2Ac) lysosomal localization (Yu et al., 2018). TRAF3IP3 positively regulates TLR4 signaling by facilitating the translocation of TLR4 into lipid rafts (Li Y. et al., 2019). In addition, TRAF3IP3 interacts with TRAF3 to regulate its localization (Dadgostar et al., 2003). We identified the NLS and NES of TRAF3IP3, which might provide a basis for its functional study in the future. In infected cells, enterovirus 3C^pro^ enters the nucleus to cleave a variety of host proteins, including transcription factors, splicing factors, and antiviral inflammatory factors, thereby interfering with host gene expression and immune responses (Flather and Semler, 2015). Our results demonstrated that TRAF3IP3 interacts with 3C^pro^ and prevents 3C-C147S from entering the nucleus to achieve antiviral effects, providing a new method by which TRAF3IP3 alters protein localization. Moreover, enteroviruses form membrane-structured replication complexes in the cytoplasm and do not enter the nucleus throughout the replication cycle (van der Linden et al., 2015). In addition to affecting 3C^pro^, TRAF3IP3 might also interfere with EV71 replication in the cytoplasm *via* other functions, which requires further investigation.

In conclusion, we identified TRAF3IP3 as a new target for 3C^pro^ and demonstrated that it can inhibit EV71 replication. EV71 3C^pro^ counteracts some of the inhibition induced by TRAF3IP3 by cleaving TRAF3IP3, representing a new example of virus-host interaction.

## Data Availability Statement

The original contributions presented in the study are included in the article/Supplementary Material, further inquiries can be directed to the corresponding author/s.

## Author Contributions

HL, YY, YC, SZ, and ZD performed the experiments. HL wrote the manuscript. HL, YY, and YC collated the data. WQ and JT provided general guidance and financial support. HL, WQ, and JT checked and approved the final manuscript. All authors contributed to the article and approved the submitted version.

## Funding

This work was supported by grants from the Key International Cooperation Project of the National Key Research and Development Program of China (grant no. 2018YFE0107600) and the Natural Science Foundation of Tianjin (grant no. 19JCZDJC35700).

## Conflict of Interest

The authors declare that the research was conducted in the absence of any commercial or financial relationships that could be construed as a potential conflict of interest.

## Publisher's Note

All claims expressed in this article are solely those of the authors and do not necessarily represent those of their affiliated organizations, or those of the publisher, the editors and the reviewers. Any product that may be evaluated in this article, or claim that may be made by its manufacturer, is not guaranteed or endorsed by the publisher.
