# Supplementary material for: TRAF3IP3 Is Cleaved by EV71 3C Protease and Exhibits Antiviral Activity
Source: Front Microbiol. 2022 Jun 23;13:914971. doi: 10.3389/fmicb.2022.914971 (PMC9260427; doi:10.3389/fmicb.2022.914971)
Supplement: Supplementary file 1 [file Table_1.DOCX]

Supplementary TABLE 1. Potential proteins that interact with EV71 3C^pro^ screened by yeast two-hybrid.

| Number | Name | Number | Name |
| --- | --- | --- | --- |
| 1 | CCP module-containing protein (AAX12481.1) | 31 | restin (EAW98323.1) |
| 2 | hCG1794476 (EAW59064.1) | 32 | Dihydropyrimidinase (NP_001376.1) |
| 3 | hCG1642725 (EAW58406.1) | 33 | hypothetical protein (CAI56716.1) |
| 4 | hCG401131 (EAW48653.1) | 34 | MDS015 (AAG14952.1) |
| 5 | hCG1990594 (EAW53972.1) | 35 | heterogeneous nuclear ribonucleoprotein L-like (NP_612403.2) |
| 6 | cytochrome b5 reductase 4 (NP_057314.2) | 36 | merosin (AAA63215.1) |
| 7 | sushi, von Willebrand factor type A, EGF and pentraxin domain-containing protein 1 precursor (NP_699197.3) | 37 | laminin alpha 2 subunit precursor variant (BAD92159.1) |
| 8 | von Willebrand factor (AAB59458.1) | 38 | la-related protein 1B (NP_001265533.1) |
| 9 | CLLL8 protein (NP_001153780.1) | 39 | protein FAM35A (KAI2556540.1) |
| 10 | SETDB2 protein (AAH47434.1) | 40 | Golgi SNAP receptor complex member 1 (NP_004862.1) |
| 11 | medium-chain specific acyl-CoA dehydrogenase (NP_001272973.1) | 41 | zinc finger MYM-type protein 2 (XP_047286555.1) |
| 12 | cysteine-rich secretory protein 2 precursor (NP_001135880.1) | 42 | CDGSH iron-sulfur domain-containing protein 2 (NP_001008389.1) |
| 13 | testis specific protein 1 variant (BAD97057.1) | 43 | zinc finger protein 198 (EAX08247.1) |
| 14 | Serine/threonine-protein kinase D3 (NP_005804.1) | 44 | Zinc finger protein 181 (AAH43228.1) |
| 15 | FCH domain only 2 (AAI37071.1) | 45 | zinc-finger protein ZBRK1 (AAG17439.1) |
| 16 | melanoma-associated antigen (AAA63235.1) | 46 | zinc finger protein 350 (NP_067645.3) |
| 17 | KIAA1641 protein (BAB13467.1) | 47 | KRAB zinc finger protein ZFQR (AAG25714.1) |
| 18 | KIAA1370 (EAW77459.1) | 48 | additional sex combs like 1 (Drosophila) (EAW76379.1) |
| 19 | ankyrin repeat domain-containing protein 36A/B (XP_016859498.1) | 49 | double-stranded RNA-binding protein Staufen homolog 2 (NP_001157855.1) |
| 20 | succinate dehydrogenase complex, subunit B (EAW94829.1) | 50 | NACP/alpha-synuclein (AAC02114.1) |
| 21 | LIM/calponin homology domains-containing protein 1 (NP_001106191.1) | 51 | KIAA0737 protein (BAA34457.3) |
| 22 | hypothetical protein (EAW92987.1) | 52 | proliferating cell nuclear antigen (NP_872590.1) |
| 23 | uncharacterized protein C9orf131 (NP_001035501.1) | 53 | FKBP15 protein (AAH77732.1) |
| 24 | bisphosphoglycerate mutase (NP_001280014.1) | 54 | Pin2-interacting protein X1 (AAK31790.1) |
| 25 | SH3 domain-binding glutamic acid-rich-like protein (NP_003013.1) | 55 | SIH002 (AAD27774.1) |
| 26 | beta-3A-adaptin subunit of the AP-3 (AAB61638.1) | 56 | TBC1 domain family, member 15 (EAW97275.1) |
| 27 | TRAF3 interacting protein 3 (EAW93441.1) | 57 | HEBP2 protein (AAH16807.1) |
| 28 | putative GTP cyclohydrolase 1 type 2 NIF3L1 (NP_068596.2) | 58 | ubiquitin-60S ribosomal protein L40 precursor (NP_001307946.1) |
| 29 | TBL1XR1 (NP_001361257.1) | 59 | ASRGL1 protein (AAH93070.1) |
| 30 | CAP-Gly domain-containing linker protein 1 (XP_005253650.1) | 60 | quinone oxidoreductase-like protein 1 (NP_665857.2) |
